# COVID‐19: vaccine’s progress

**DOI:** 10.1111/1751-7915.13818

**Published:** 2021-05-07

**Authors:** Harald Brüssow

**Affiliations:** ^1^ Department of Biosystems Laboratory of Gene Technology KU Leuven Leuven Belgium

## Abstract

Under the pressure of the COVID‐19 pandemic, vaccines were developed and rolled out into mass vaccination campaigns at incredible speed. What normally takes a decade was worked out within a year. Vaccines were produced along many different platforms ranging from inactivated whole virus vaccines over adenovirus‐vectored vaccines, recombinant protein vaccines and nanoparticles to mRNA vaccines. Several vaccines went through preclinical testing and completed successful phase 1 to phase 3 clinical trials. The first evaluations of national vaccination campaigns document astonishing high levels of protection against disease. The present article summarizes the published reports leading to these striking achievements with vaccines based on different concepts.

‘This is a triumph. Most vaccines have taken decades to develop, but this one is likely to move from conception to large‐scale implementation within a year’. These are the enthusiastic words of the editors of *The New England Journal of Medicine* when commenting on the outcome of a phase 3 mRNA vaccine trial against COVID‐19 (Rubin and Longo, 2020). A comment in *Science* was less enthusiastic about the AstraZeneca adenovirus‐vectored SARS‐CoV‐2 vaccine trial mentioning the blending of two trial results and the dosing error that led to the puzzling result that a lower dose led to a better efficacy. However, this criticism addresses more shortcomings of the planning and execution of the clinical trials than a critical attitude towards the adenovirus vaccine platform (Cohen, 2020; Ledford 2020). As reported later in this minireview, large vaccination campaigns showed the comparable efficacy of both vaccine concepts. The adenovirus‐based COVID‐19 vaccine is with costs of about $3 per dose cheaper than the mRNA vaccines which will cost at least $20 per dose, is less demanding with respect to storage and has the potential to be protective with a single injection. However, major strides forward have also been made with other vaccine platforms ranging from classical approaches with inactivated viruses to futuristic approaches with pan‐corona nanoparticle vaccines. Before COVID‐19 vaccines became available, the World Health Organization (WHO) defined valuable vaccines as those showing a > 50% efficacy, and nobody would have predicted that we would get vaccines that showed in phase 3 trials efficacy values exceeding 90%. This Lilliput minireview summarizes recent progress in the COVID‐19 vaccine field.

## Inactivated whole viruses

The inactivated whole‐virus vaccine platform is an established manufacturing platform for vaccine production, and aluminium salts added to these viruses are the most commonly used adjuvants used in human vaccines, but frequently require multiple doses for inducing protection. Because of the classical concept, the ease of production and upscaling, low regulatory hurdles and the relatively low cost, inactivated vaccines can capture worldwide a sizeable portion of the COVID‐19 vaccine market. There are many inactivated vaccines at various clinical stages. Major differences are the kind of chemical inactivation and additional adjuvantia (Iversen and Bavari, 2021).

### BBIBP vaccine from China

More than 600 participants received the inactivated virus vaccine BBIBP‐CorV produced by the Beijing Institute of Biological Products. In a first phase, different doses of 2, 4 or 8 μg virus were injected, which showed seroconversion rates of 79%, 87% and 96% respectively. Participants older than 60 years showed a slower increase in serum antibodies. In a second phase, either a single 8 μg dose or two doses of 4 μg were given 2, 3 or 4 weeks apart. Recipients of two 4 μg doses showed higher neutralizing antibody titres than the recipients of a single 8 μg dose. No significant difference was seen for neutralizing antibodies with respect to different timing between the two doses (Xia et al., 2021).

### Sinovac vaccine

Sinovac Life Sciences (Beijing) tested 3 and 6 μg CoronaVac virus given in two injections separated by 2 and 4 weeks. In a phase 1 trial, a seroconversion rate of only 50% was seen for neutralizing antibodies in the day 0/14 schedule, while 80% seroconversion was achieved with the day 0/28 schedule. In a phase 2 trial, greater than 90% seroconversion was seen with both doses and with both vaccination schedules. The scientists attributed the difference to vaccine production in a cell culture or bioreactor system respectively. The bioreactor‐produced vaccine had indeed twice as much intact spike protein on the virus surface than the cell culture‐produced virus. Mean neutralizing titres were lower than those of convalescent sera. T‐cell responses measured by ELISpot were low (Zhang et al., 2021). CoronaVac was then tested in subjects older than 60 years who received either 1.5, 3 or 6 μg inactivated vaccine with alum adjuvant or placebo (adjuvant only) twice by intramuscular injection, separated by 28 days. 21% of the 420 participants experienced an adverse event which consisted of injection site pain (9%) and fever (3%). A serious adverse event of pancreatitis occurred but was considered unrelated to the vaccine. Seroconversion was 50–60% after the first injection and 90–100% after the second injection. Mean neutralizing titres were low after the first injection but increased after the boost injection (Wu et al., 2021). According to press releases, phase 3 trial data from Turkey indicated a 91 % efficacy at preventing symptomatic disease on the basis of 29 observed COVID‐19 cases among 1322 volunteers. Results from an efficacy trial of about 1600 people in Indonesia found the vaccine was 65% effective at preventing symptomatic disease on the basis of 25 observed COVID‐19 cases. The Brazil trial recorded 252 cases of COVID‐19 – 167 in people who received the placebo and 85 who were vaccinated – when tested in 9,200 healthcare workers suggesting 50% vaccine efficacy at preventing severe and mild COVID‐19 (Mallapaty, 2021).

### Sinopharm vaccine

According to a press release, efficacy of the two‐dose inactivated vaccine from the Chinese company Sinopharm was 86% in a phase III trial in 31 000 people in the United Arab Emirates. Sinopharm claims that it can produce billions of doses and has vaccine orders from more than 100 countries. Scientists worry that some countries have to choose between accepting a vaccine without published scientific analysis or not getting a vaccine since vaccine procurement is currently a problem in the EU (Cyranoski, 2020).

### BBV152 vaccine from India

BBV152, manufactured by Bharat Biotech / India, is a whole‐virion β‐propiolactone‐inactivated SARS‐CoV‐2 vaccine displaying the 614G variant mutation, propagated on Vero cells. The vaccine is formulated with a toll‐like receptor 7/8 (TLR 7/8) agonist molecule (imidazoquinoline) adsorbed to alum (Algel). Adverse reactions were reported in 10 to 20% of the participants and consisted mainly of injection site pain (5%), headache (3%), fatigue (3%) or fever (2%). Neutralizing antibody titres were comparable to those seen in convalescent sera of COVID‐19 patients. CD4+ and CD8+ T‐cell responses to the virus were detected (Ella et al., 2021a). The vaccine was then tested at doses of 3 and 6 μg given to 380 healthy adults and adolescents in India, applied by two intramuscular injections, separated by 4 weeks. Mean titres were twofold higher with the higher vaccine dose, and neutralizing antibody titres were comparable to those in convalescent sera of COVID‐19 patients. The seroconversion rate ranged from 88% to 98%. The vaccine elicited CD4+ and CD8+ T‐cell responses to the virus displaying a T helper type 1 (Th1) phenotype, making enhancing antibodies less likely (Ella et al., 2021b,2021a). The 6 μg dose of this vaccine, which can be stored between 2 and 8°C, entered now a phase 3 clinical trial with 25 000 participants but has already received emergency use authorization in India. After challenge with wild‐type SARS‐CoV‐2, placebo receiving rhesus monkeys showed high viral load in the nose, throat and lung and displayed pronounced lung pathology. In contrast, the vaccinated animals showed substantially reduced nasal viral load and no virus in the throat and in the lungs and no lung pathology (Yadav et al., 2021).

## Adenovirus‐vectored vaccine

A number of research institutes and companies used recombinant adenoviruses as vaccine carrier. The adenovirus was modified such that it cannot initiate a productive viral infection. The carrier virus enters cells, expresses the SARS‐CoV‐2 spike protein and then stops the virus lifecycle due to a replication block. The vaccine‐infected cells are destroyed by the very immunity they elicit. Adenovirus vectors do not need an adjuvant but require high doses typically of 10^10^ or 10^11^ particles per injection.

### Oxford‐Astra Zeneca vaccine

A total of 560 subjects in three age groups (18–55, 56–69 and 70 years and older) received either one or two intramuscular injections of the chimpanzee adenovirus‐vectored vaccine ChAdOx1 nCoV‐19 (at 2× or 5 × 10^10^ virus particles) expressing the SARS‐CoV‐2 spike protein. A chimpanzee adenovirus vector was chosen to avoid interference with a pre‐existing immune reaction against human adenovirus from previous natural infections. Controls received a meningococcal protein‐polysaccharide conjugate vaccine. Adverse events (injection‐site pain, feeling feverish, muscle ache and headache) decreased with the age of the subjects from 88% to 73% and 61% respectively. Subjects receiving only one dose had about fivefold lower IgG and neutralizing antibody titres than those receiving a prime and booster dose. No significant difference was seen in neutralization titres between low‐dose and standard‐dose vaccine recipients. Neutralizing antibody titres after a boost dose were similar across all age groups. By 14 days after the boost dose, 99% of boosted participants had neutralizing antibody responses, and reached titres of 200–400. Cellular IFN‐γ ELISpot responses against SARS‐CoV‐2 spike protein peaked 14 days after the prime vaccination and did not increase significantly after the boost vaccination (Ramasamy et al., 2021).

Interim analysis for three pooled phase 3 clinical trials conducted in the UK and Brazil with 23 848 participants was subsequently published. The participants were randomized on a 1:1 basis to ChAdOx1 nCoV‐19 at a standard dose (SD) of 5 × 10^10^ viral particles given in two injections, separated by a median of 2 months. A meningococcal vaccine served as control. Many participants were health care workers from UK and Brazil; they were predominantly white (UK: 91%; Brazil: 66%), and only 2% were older than 70 years. Reactions to the vaccine were comparable to the licensed meningococcal vaccine. In a parallel test conducted in the United States, three myelitis cases (two in adenovirus vaccine group, one was an undiagnosed multiple sclerosis case) were observed, which led to temporary recruitment stop in the trial. The primary end‐point was occurrence of clinically and virologically diagnosed COVID‐19. An overall vaccine efficacy of 70% was observed in that interim analysis: 0.5% of adenovirus vaccine recipients developed symptomatic disease vs. 1.7% in the meningococcal vaccine control group (Voysey et al., 2021a). Severe COVID‐19 counted from 21 days after the 1^st^ dose occurred in none of the adenovirus recipients, compared with five severe cases in controls. By a virus titration error, subjects of the first UK trial received only half as much virus (unintended low dose, LD) as first injection and showed unexpectedly a 90% vaccine efficacy compared with the 60–64% efficacy with the planned SD doses in UK and Brazil respectively. In the UK trial, weekly self‐swabs were assessed for the presence of viral RNA. Infections without documented clinical symptoms were seen in 0.6% participants receiving the LD/SD scheme vs. 1.5% in controls demonstrating a 59% vaccine efficacy against nasopharyngeal viral replication, while no difference was seen in the SD/SD recipients compared with controls. The authors admit limitations of their study: few old participants or subjects with comorbidity were enrolled and the reported results represent an interim analysis of three pooled ongoing trials (Voysey et al., 2021a). Immunologists argued that the higher protection rate achieved with the lower dose might find an explanation by a quicker establishment of memory immune cells or less interference by antibodies raised against the chimpanzee part of the adenovirus vector (Callaway, 2020).

An update of the pooled clinical trials conducted in UK, Brazil and South Africa was published with efficacy estimation based on 332 symptomatic COVID‐19 cases, instead of 131 cases evaluated in the first interim analysis. In the vaccine group, 1.0% experienced a symptomatic infection compared with 2.9% in the control group, demonstrating an efficacy of 67%. Vaccine efficacy against COVID‐19 cases requiring hospital admission was 100% (0 vs. 15 cases). The studies were initially planned as single‐dose studies but were amended to incorporate a second dose after review of insufficient immunogenicity in a phase 1 trial. However, some participants chose not to receive the second dose. Due to vaccine production problems, there were in addition delays in administration of the second dose for a large number of trial participants. These peculiar conditions allowed to explore the effect of the time interval between prime and boost injection or of a single injection. Vaccine efficacy after two standard doses was 55% when given with an interval of < 6 weeks and 81% when the two injections were given more than 12 weeks apart. A single injection provided protection against primary symptomatic COVID‐19 in the first 90 days with an efficacy of 76% but was not efficacious against asymptomatic infection. Antibody titres decreased by 34% at day 90 and by 64% at day 180 after vaccination. Plotting anti‐SARS‐CoV‐2 spike IgG titres against vaccine efficacy for each dose interval showed a linear relationship, suggesting antibodies as a potential correlate of protection (Voysey et al., 2021b).

### Gamaleya‐Sputnik V vaccine

Russian scientists from the Gamaleya Institute tested a prime injection with recombinant adenovirus type 26 (rAd26) vector followed 3 weeks later by a boost injection with recombinant adenovirus type 5 (rAd5) vector, both expressed the SARS‐CoV‐2 spike S glycoprotein. They tested vaccines stored frozen (−18°C) or lyophilized vaccine allowing storage at 2 to 8°C in 76 volunteers. Adverse events were seen, but mild and transient and typical for adenovirus‐vectored vaccines. Seroconversion rate was 100%, and neutralizing antibody titres were comparable to those in convalescent COVID‐19 patients; ELISA anti‐spike IgG titter was 10‐fold higher than after infection. Prime‐boost immunization was superior to single‐dose immunization, which led only to low antiviral titres. Formation of antigen‐specific cells of both T helper (CD4+) and T‐killer (CD8+) cells, and of interferon‐γ secretion in peripheral blood mononuclear cells was seen in 100% of volunteers. Pre‐immune serum antibody titres to the adenovirus vectors were low at baseline, increased after vaccination, but did not interfere with antibody development against the SARS‐CoV‐2 S protein (Logunov et al., 2020).

This Russian Gam‐COVID‐Vac (Sputnik V) vaccine was tested among 22 000 adults in 25 hospitals from Moscow. The participants were randomly assigned (3:1) to receive frozen vaccine or placebo. The vaccine was administered intramuscularly in a prime‐boost regimen with a 21‐day spacing. The primary outcome was the proportion of participants with PCR‐confirmed symptomatic COVID‐19 counted from day 21 after receiving the first dose. The most common adverse events were flu‐like illness, injection site reactions, headache and asthenia. Serious adverse events were noted in 0.3% of the vaccine and in 0.4% of the placebo group. Three deaths occurred in the vaccine group, one in the placebo group. Two deaths in the vaccine group were associated with COVID‐19. Their COVID‐19 symptoms occurred 4–5 days after first vaccine dose and represent therefore undiscovered infections acquired before vaccination. Both patients had comorbidities and self‐administered non‐steroidal anti‐inflammatory drugs without informing the clinicians. As of 23 January 2021, more than 2 million doses of Gam‐COVID‐Vac have already been administered to the public, controlled by the Federal Service for Surveillance in Healthcare.

In the vaccine group, 98% and 96% of the subjects seroconverted for virus spike‐specific IgG and for neutralizing antibodies, respectively, compared with 15% and 7% in the placebo group. Only 2% of the vaccinees were non‐responders. Notably, 11% of the subjects were older than 60 years and they showed a good humoral immune response. Cellular immune response (secretion of IFN‐γ by peripheral blood mononuclear cells stimulated with the viral spike protein) occurred in 98% of the tested vaccinees and in 0% of the placebo recipients. During the first 2 weeks after the first dose, there was no significant difference in incidence of COVID‐19 cases between the vaccine and placebo groups. From day 15 to 21 after the first dose, efficacy was 74%, and from day 21 100%, giving an overall vaccine efficacy of 91% in this interim analysis based on 78 observed COVID‐19 cases (Logunov et al., 2021).

### Johnson & Johnson vaccine

Adenovirus Ad26 vector expressing a full‐length, stabilized SARS‐CoV‐2 spike protein developed by Janssen Vaccines was tested in 400 adults from Belgium and the United States. High‐grade fever was noted in < 10% of the vaccinees and resolved within 1 or 2 days. ELISA antibodies to the spike protein increased in 99% of the vaccine recipients, and antibody titres were modestly higher with a higher dose. ELISA antibodies were modestly higher in those receiving two instead of one injection, and neutralizing antibody titres were only fivefold higher in subjects receiving two instead of one vaccine shots. Antibody titres were only marginally lower in older vaccine recipients. A CD4+ Th1‐biased response to S peptides was detected in 76% of the low‐dose and in 83% of the high‐dose recipients, with a slightly lower rate in older subjects. A CD8+ T‐cell response was detected in 51% of low‐dose and in 64% high‐dose recipients, with a significantly lower percentage in older subjects. Neutralizing antibody titres against the Ad26 vector were elicited by the first vaccination, but did not correlate with the magnitude of immune response after the second dose. Notably, a single dose of the adenovirus vaccine elicited a strong humoral response in 90% of vaccine recipients, regardless of age or vaccine dose, which represents an obvious logistic advantage over a two‐dose vaccine, especially in developing countries (Sadoff et al., 2021). Johnson & Johnson, the US pharmaceutical company developing the Janssen vaccine, communicated in a press release that its vaccine was 66% effective in global trials. Vaccine efficacy was 72% in trials conducted in America (The Economist, 2021, 30th January).

### Mucosal adenovirus vaccines

All SARS‐CoV‐2 vaccines in current clinical tests are injected vaccines. A viral infection starting in the nasopharynx might however best be prevented by a mucosal, more specifically a nasal vaccine. Chinese virologists engineered an adenovirus, Ad5‐S‐nb2, carrying a codon‐optimized SARS‐CoV‐2 spike gene and explored it after intramuscular (IM) or intranasal (IN) application. IM vaccination induced a good serum ELISA and neutralizing antibody response, but no mucosal antibodies while IN vaccination induced both serum and mucosal antibody titres. Serum antibody titres increased over time in IM injected, but not in IN immunized animals resulting in 10–100 fold higher titres in IM than in IN immunized animals. IN vaccination also induced a weaker cell‐mediated immune response to the spike protein than IM vaccination. Despite these observations, IN vaccination conferred effective protection of monkeys against SARS‐CoV‐2 infection. IN induced less anti‐vector antibodies than IM immunization which is a possible advantage for repeat immunizations if the same adenovirus vector vaccine has to be used (Feng et al., 2020). US researchers had previously reported similar promising data for intranasal immunization of mice with a chimpanzee adenovirus vector expressing the SARS‐CoV‐2 spike protein (Hassan et al., 2020) and have now extended their observations to rhesus monkeys. Rhesus macaques were immunized with ChAd‐Control or ChAd‐SARS‐CoV‐2‐S and challenged one month later by combined intranasal and intrabronchial inoculation with SARS‐CoV‐2. A single intranasal dose of ChAd‐SARS‐CoV‐2‐S induced neutralizing antibodies and T‐cell responses and limited or prevented infection in the upper and lower respiratory tract, respectively, after SARS‐CoV‐2 challenge (Hassan et al., 2021).

## Alternative experimental viral vectors

### Yellow fever virus

The flavivirus YF17D is a live attenuated yellow fever virus vaccine, which rapidly induces broad multifunctional innate, humoral and cell‐mediated immune responses that result in lifelong protection after a single vaccine dose. Researchers from KU Leuven (Belgium) introduced the SARS‐CoV‐2 spike protein gene in different molecular forms into this licensed vaccine. In a hamster model, the vector expressing the uncleavable S0 protein induced neutralizing antibodies with high titres. The vaccinated hamsters were protected against a subsequent intranasal viral challenge by sterilizing immunity. Vaccine response in mice indicated a dominant pro‐inflammatory antiviral T helper 1 (TH1) cell polarization of the immune response and the presence of S‐specific interferon gamma (IFNγ)‐expressing CD8+ and CD4+ T lymphocytes. The cleavable S1 /S2 form of the spike protein in the vaccine induced a less good protection, while the S1 subdomain in the vaccine vector induced no neutralizing antibodies, albeit a cellular immune response, which conferred however no protection. Macaques vaccinated with the S0 form mounted a strong neutralizing antibody response and were protected from challenge with wild‐type SARS‐CoV‐2. Such a vaccine has the potential to protect against both COVID‐19 and yellow fever, which is attractive asset for equatorial Africa and America (Sanchez‐Felipe et al., 2021).

### Measles virus

The live attenuated measles virus (MeV) vaccine is one of the safest and most efficient human vaccines, which induces lifelong immunity. It is also a highly immunogenic recombinant virus vector which was previously used to express numerous foreign viral antigens in adults, even against pre‐existing anti‐MeV vector immunity. The stabilized prefusion S protein (rMeV‐preS), in which the furin cleavage site was deleted, induced more neutralizing antibodies against SARS‐CoV‐2 than vectors expressing only the receptor‐binding domain (RBD). This vector provided complete protection of hamsters against challenge with SARS‐CoV‐2, preventing virus replication in lungs and nasal turbinates, body weight loss, cytokine storm and lung pathology (Lu et al., 2021).

### Vaccinia virus

Another popular vector is the Modified Vaccinia virus Ankara (MVA). It was attenuated by 500 passages in chicken embryo fibroblasts rendering it incapable to replicate in human cells. The vector itself is a licensed smallpox vaccine, but can also efficiently express foreign proteins. The SARS‐CoV‐2 S gene was cloned into this vector and immunity induced by a single vaccination of mice prevented morbidity after challenge with SARS‐CoV‐2. Viral RNA synthesis in the lungs was suppressed, and cytokine production was greatly reduced. Only low amounts of virus were found in the nasal turbinates, indicating a potential for protection of the upper respiratory tract (Liu et al., 2021).

## Recombinant protein vaccines

### Novavax vaccine

The US company Novavax has accumulated safety data on 14 000 participants in various nanoparticle vaccine trials, including children, pregnant women and on 4300 participants exposed to Matrix‐M1 adjuvant, a saponin‐based adjuvant. In the current vaccine, this adjuvant is mixed just before injection with a recombinant SARS‐CoV‐2 spike glycoprotein in a prefusion conformation. The protein was produced by baculovirus in an insect cell culture system. Structural biologists investigated the recombinant spike protein in the presence of the adjuvant by cryo‐electron microscopy. Strikingly, all three RBDs of the spike trimer were in the closed conformation; however, ELISA showed that the recombinant protein binds to the host receptor ACE2, indicating that the RBD is in a dynamic state and accessible. The EM images showed free trimers and multi‐trimer rosettes, arranged around a micellar core of detergent and cage structures (Bangaru et al., 2020).

Mice receiving the protein vaccine with adjuvant showed upon viral challenge no virus in the lung, while protein vaccine without adjuvant reduced the viral titre only by 10‐fold compared with placebo receiving mice. Vaccine with adjuvant protected mice from weight loss and attenuated lung histopathology. IFN‐γ +, TNF‐α + and IL‐2 + cytokine‐secreting CD4+ and CD8+ T cells were significantly higher in mice receiving the protein vaccine with adjuvant than without adjuvant. CD4+ T follicular helper (Tfh) and germinal centre (GC) B 277 cells in spleen were increased in immunized mice indicating a strong immune response. In baboons, high neutralizing titres were observed after a prime/boost immunization with the vaccine protein plus adjuvant with titres of 6400–17 000 while animals receiving the protein vaccine without adjuvant had little or no detectable neutralizing antibodies which strongly supports the role of the saponin adjuvant in promoting the generation of neutralizing antibodies (Tian et al., 2021).

In human safety studies, adverse events were absent or mild (pain at injection site, fatigue, headache), more common with adjuvant and after the second injection, but of short duration. Virus‐specific antibodies were significantly higher after two than one injection and significantly higher than in convalescent sera from COVID‐19 patients when given with the adjuvant, irrespective whether 5 or 25 μg of antigen was injected. ELISA and neutralizing antibodies showed a close correlation. CD4+ T‐cell responses with IFN‐γ, IL‐2 and TNF‐α production on spike protein stimulation indicated a Th1 phenotype while Th2 responses were minimal, making enhancing antibody production unlikely (Keech et al., 2020).

At a press conference, Novavax communicated data from two efficacy trials (Callaway and Mallapaty, 2021). The UK trial enrolled 15 000 participants, half received the recombinant protein vaccine, half a placebo. The investigators detected 6 infections in the vaccine group, versus 57 in the placebo arm, indicating an efficacy of 89%. The UK trial took place when the fast‐spreading variant, known as B.1.1.7, was taking hold. The South Africa trial enrolled 4,400 participants. The 501Y.V2 variant now accounts for more than 90% of COVID‐19 cases in South Africa and also in that trial. The group receiving two recombinant protein vaccine doses recorded 15 COVID‐19 cases, compared with 29 cases among participants who received a placebo, suggesting an efficacy of 49%. Notably, within the placebo group, COVID‐19 infections were as likely in participants who had SARS‐CoV‐2‐specific serum antibodies from natural infection experienced during the first wave of the pandemic or in seronegative participants, suggesting immune evasion of the variant virus being responsible for the lower protection rate in the South African trial.

### Sanofi‐GSK vaccine

Sanofi’s recombinant technology and GlaxoSmithKline’s pandemic adjuvant are established vaccine platforms that have proven successful against influenza virus. In a December 2020 press release they announced a delay in their COVID‐19 vaccine development due to problems with a weak immune response in older adults to their adjuvanted recombinant protein COVID‐19 vaccine. (GSK Press Release, 2020).

### Clover Biopharmaceuticals vaccine

Clover Biopharmaceuticals (China) produced a recombinant SARS‐CoV‐2 spike protein linked to human type I procollagen, which leads to self‐trimerization of the fusion protein via disulphide bonds. The protein called SCB‐2019 was produced in mammalian cell culture. The protein is at 2–8°C stable for 6 months. The vaccine candidate was tested in Australian volunteers at different doses and in different formulations. Vaccination was well tolerated: systemic adverse events were more frequent in younger adults (38%) than in older adults (17%), and at the first compared with the second injection and consisted of headache, fatigue and myalgia, which resolved spontaneously. Anti‐spike IgG antibodies did not increase after the first dose of non‐adjuvanted SCB‐2019 by day 22, irrespective of dose level and few subjects seroconverted after the second injection. In contrast, about 90% of the vaccinees seroconverted after injection of the adjuvated vaccines with IgG antibody titres 2‐ to 6‐times higher than in convalescent sera from COVID‐19 patients. In participants receiving the oil‐in‐water emulsion adjuvant AS03, titres were not much dose‐ and age‐dependent. AS03 was used in previous pandemic H5N1 influenza vaccines. When the fusion protein was given with the dinucleotide CpG and alum adjuvants, antiviral titres were overall lower (particularly in older subjects) and antigen dose‐dependent. The researchers observed a strong correlation between neutralizing activity and IgG activity in virus binding tests. In both adjuvanted vaccine groups, but not in the non‐adjuvanted group, increases in IFN‐γ‐positive and IL‐2‐positive CD4+ T cells were observed indicative of a T helper (Th)‐1‐biased cell‐mediated immune response. The 9 µg SCB‐2019 adjuvanted with AS03 and 30 µg SCB‐2019 adjuvanted with CpG/Alum are now in a phase 2/3 trial (Richmond et al., 2021).

### RBD vaccine

Chinese scientists developed a recombinant protein vaccine consisting of the RBD of the spike protein coupled to a signal peptide in order to ensure protein secretion in an insect cell culture system. In animals, a single injection of the recombinant protein showed an ELISA antibody response after 7 days that was dose‐dependent and increased by use of aluminium hydroxide gel adjuvant, and by boost injections. The researchers immunized non‐human primates with two intramuscular injections of 20 μg protein antigen on days 0 and 7 and then challenged the monkeys with live SARS‐CoV‐2 viruses 28 days later. Viral replication was not detected in the lung, throat or anal swabs of the challenged immunized monkeys. The histology of the lung remained normal. The lymphocytes isolated from vaccine‐immunized mice induced increased levels of interferon IFNγ and interleukin IL‐4 when stimulated with the recombinant RBD, but adoptive transfer of splenic T cells (CD4+ and CD8+ cells) did not provide protection against infection with SARS‐CoV‐2. By contrast, adoptive transfer of 0.1 ml of the pooled sera from immunized mice prevented viral replication in challenged mice (Yang et al., 2020).

Australian researchers raised the question whether the whole spike protein represents a better antigen than just the receptor‐binding domain (RBD) of the spike protein S. This is clearly the case in mice, because T follicular helper (TFH) cells are less induced by RBD than by S. In contrast, both S and RBD vaccines were comparably immunogenic in macaques. Heterologous antigen use with spike for priming and RBD for boost injection has the potential to focus the immune response on RBD epitopes (Tan et al., 2021).

### Protein vaccine engineering

The prefusion conformation increases the immunogenicity of the spike protein. However, such proteins have the tendency to adopt the more stable post‐fusion conformation, which can aggregate and thus decrease antigen yield. US researchers designed amino acid substitutions to stabilize the prefusion form by disulphide bonds and introduced salt bridges to neutralize charge imbalances, or hydrophobic residues to fill internal cavities, and prolines to stabilize loops. Combining six proline substitutions gave higher expression and allowed storage at room temperature and resistance against three freeze‐thaw cycles (Hsieh et al., 2020).

### Nanoparticle vaccines

US researchers engineered nanoparticle consisting of a 28 nm‐wide, 120‐subunit complexes with icosahedral symmetry constructed from trimeric and pentameric components, which assembled in vitro by simply mixing yielding a structure resembling an empty viral capsid. The RBD of the spike protein was added to the more numerous trimeric protein subunits via a protein linker exposing the viral antigen at the capsid surface. The recombinant RBD‐trimer protein was expressed in a mammalian cell culture system to obtain a glycosylated viral RBD. Immunization of mice with RBD nanoparticles resulted in an expansion of RBD‐specific B cells and germinal centre (GC) precursors and B cells with class‐switched RBD‐specific B cells. The nanoparticles can co‐display multiple antigens and thus improve the breadth of vaccine‐elicited immune responses. The nanoparticles resemble virus‐like particle vaccines, which are already registered vaccines for human papillomavirus and hepatitis B virus that provide potent, durable immunity even after a single vaccination (Walls et al., 2020).

### Pan‐corona nanoparticle vaccines

A US and UK research consortium worked with virus‐like particles where the outer shell viral proteins contain a catcher sequence, which form spontaneous isopeptide bonds with purified antigens containing a 13‐residue tag sequence. These nanoparticles allow an efficient ‘plug and display’ strategy. Multivalent display of antigen enhances B‐cell responses and can provide longer‐lasting immunity. They added RBD from four to eight different beta‐coronaviruses, which are potential sources for novel zoonoses. After a single injection such mosaic‐RBD‐nanoparticles elicited in mice antibodies with cross‐reactivity to heterologous RBDs without compromising the response to the homotypic SARS‐CoV‐2 RBD. Such vaccine candidates are attractive for application in animals to pre‐empt the spillover of further animal coronaviruses from bats or minks into the human population (Cohen et al., 2021).

## mRNA vaccines

### Pfizer‐BioNTech: Phase 1/2 studies

The German company BioNTech conducted with the support of Pfizer and US scientists a phase 1/2 vaccine study: 45 healthy adults received two doses, separated by 21 days, of 10, 30 or 100 µg mRNA. The mRNA in this trial encoded only the RBD of the SARS‐CoV‐2 spike protein fused to the bacteriophage T4 fibritin‐derived fold on trimerization domain to increase its immunogenicity by multivalent display. Base replacement with pseudouridine dampened innate immune sensing and increased mRNA translation in vivo. Pain at the injection site was the dominant symptom, followed by fatigue, headache and fever, which generally resolved within one day. Transient decreases in lymphocyte counts were seen since RNA vaccines are known to induce type I interferon, which is associated with transient migration of lymphocytes into tissues. Reactogenicity was dose‐dependent and greater after the second injection, leading to the suppression of the second 100 µg mRNA injection from the protocol. RBD‐binding IgG ranged from 500 to 1800 after the first dose (convalescent sera showed titres of 600) and increased to 5000–28 000 after the second injection. Neutralizing antibody titres were modest after one dose, but reached titres of 180 and 440 for the second dose with 10 µg and 30 µg mRNA doses, respectively and compared favourably with convalescent sera from COVID‐19 patients that displayed a titre of 90. RBD IgG titres decreased, but neutralizing titres continued to rise up to 35 days post‐injection (Mulligan et al., 2020).

A second phase 1/2 trial was conducted in Germany: 60 healthy volunteers younger than 55 years received lipid nanoparticle‐formulated mRNA vaccine encoding RBD in doses varying from 1 to 60 μg mRNA. Reactogenicity was dose‐dependent and stronger than in the previous US trial, but transient (local tenderness, increased inflammation markers). A strong, dose‐dependent vaccine‐induced antibody response against RBD and neutralizing antibodies against the virus were seen after the boost injection while only a weak response was displayed after prime immunization. However, 3 weeks after the boost, the antibody titres started to decrease. 95% of vaccinees mounted RBD‐specific CD4+ T‐cell responses which correlated with neutralizing antibody titres and also needed a boost immunization. CD8+ T‐cell responses were seen in 76% of the vaccinees, they did not correlate with antibody titres and were with the 1 μg dose as good as with higher doses. Cytokines secreted in response to stimulation comprised IFNγ, TNF, IL‐1β, but not IL‐4 or IL‐5 indicating a favourable Th1 profile. A variant of this mRNA vaccine which encodes the entire spike protein was compared to the RBD mRNA vaccine in 200 adults, ranging in age from 18 to 85 years. Both mRNA vaccines showed in young and old vaccinees comparable neutralizing antibody titres to those of convalescent sera. However, the full‐length mRNA vaccine showed a lesser amount of adverse events particularly in older individuals (Walsh et al., 2020a,2020b).

Pfizer/ BioNTech then reported on two lipid nanoparticle‐formulated, nucleoside‐modified mRNA vaccine candidates: BNT162b1, which encodes a secreted trimerized SARS‐CoV‐2 receptor‐binding domain (RBD); or BNT162b2, which encodes a membrane‐anchored SARS‐CoV‐2 full‐length spike protein, stabilized in the prefusion conformation. Vaccine at the different doses or placebo was applied in a factorial design to 195 healthy volunteers in the US in two age groups (18–55 and 65–85 years). The 100 μg dose was abandoned after the first injection when observing high reactogenicity. Systemic reactions were fever and chills, which were higher after the second injection (75% of the participants showed 38°C fever). Systemic response to mRNA b2 was milder than to mRNA b1. Reactions were milder in older than in younger vaccinees. Lower neutralizing antibody titres were seen in 65‐ to 85‐year‐old compared with 18‐ to 55‐year‐old participants, but in all age groups neutralizing titres were as high or up to five times higher than those from a convalescent serum panel of COVID‐19 patients (Walsh et al., 2020a,2020b).

### Pfizer‐BioNTech: Phase 3 trial

BNT162b2 mRNA vaccine was then tested in a phase three trial with 43 548 participants randomized on a 1:1 basis to 30 μg mRNA or saline placebo for two intramuscular injections, separated by 21 days. The subjects came from six different countries, but the majority were from the United States (70%) and were whites (83%). Their median age was 52 y (range 16‐91 years); and 35% were obese. As side reactions, fever was less prevalent in older than in younger participants. Side‐effects were transient and mild, necessitating only antipyretic and pain medication. The primary efficacy outcome was COVID‐19 occurrence defined by typical clinical symptoms and a positive RT‐PCR test. When counted from day 7 after the 2nd vaccine dose, there were nine cases in the vaccine and 172 in the placebo group, demonstrating a vaccine efficacy of 94.8%. The vaccine and placebo groups showed until day 10 the same case count, but already two weeks after the 1st injection, a 50% vaccine protection was achieved, while after the second injection, practically no new infections were seen in the vaccine group. A similar vaccine efficacy was seen across all subgroups, including 1500 participants older than 75 years. The vaccine seems also to protect against severe disease (one in vaccine, nine in placebo group) (Polack et al., 2020).

### Pfizer‐BioNTech: Impact on viral infections

Pfizer has not incorporated regular nasal swabbing into the phase 3 clinical trial protocol, and they could thus not verify whether the vaccine reduces the rate of infections and thereby decrease the rate of virus transmission.

British researchers analysed the occurrence of positive viral PCR tests in healthcare workers (HCW) during two weeks in January 2021. The HCW had no symptoms and half of them had received the first dose of the Pfizer BioNTech mRNA vaccine. 26 of 3252 (0.80%) tests from unvaccinated HCWs were positive, compared with 13 of 3535 (0.37%) from HCWs <12 days post‐vaccination and 4 of 1989 (0.20%) tests from HCWs ≥12 days post‐vaccination. These numbers suggest a significant fourfold decrease in the risk of asymptomatic SARS‐CoV‐2 infection among HCWs ≥12 days post‐vaccination compared with unvaccinated controls (Weekes et al., 2021).

### Moderna mRNA vaccine: preclinical data

Moderna’s mRNA vaccine encodes a stabilized SARS‐CoV‐2 spike protein in a pre‐fusion conformation which elicited in mice a better pseudovirus neutralizing antibody responses than vaccination with the secreted spike protein. Two doses of 1 μg RNA vaccine application rendered viral replication in the nose undetectable in 6 out of 7 mice. A single dose of 1 µg of mRNA1273 completely protected against lung viral replication. Importantly, sub‐protective 0.1 and 0.01 µg mRNA doses showed no evidence of enhanced lung pathology making a vaccine‐associated enhanced respiratory disease a lesser concern (Corbett et al., 2020).

### Moderna: early clinical trials

In a phase 1 dose‐escalation trial, 40 older adults were stratified according to age (56 to 70 years or ≥ 71 years). Adverse events included fatigue, chills, headache and myalgia. Adverse events were dose‐dependent and were more prominent after the second immunization. ELISA antibodies against the viral spike protein were also dose‐dependent, antibodies increased already with the first injection and showed further increases after the second injection when titres were comparable or superior to convalescent sera from COVID‐19 patients. Neutralizing antibody titres did not differ between the age groups. The vaccine elicited a strong CD4 cytokine response involving type 1 helper T (Th1) cells in all vaccinees and showed a minimal Th2 response (Anderson et al., 2020).

Next, 34 healthy adults received 100 μg of Moderna’s mRNA vaccine in two injections separated by 28 days and were investigated for serum antibodies in serial blood samples collected over 4 months after the first injection. ELISA antibodies increased to high titres that showed only a minimal decline over time. Serum neutralizing antibody titres against SARS‐CoV‐2 were higher than the titres from convalescent sera of COVID‐19 patients one month after symptom development (Widge et al., 2021).

### Moderna: efficacy trial

In July 2020, 30 420 volunteers were randomly assigned in a 1:1 ratio to receive either Moderna’s vaccine (100 μg mRNA encoding the prefusion‐stabilized spike glycoprotein in a lipid nanoparticle‐encapsulated preparation) or placebo (saline) twice by intramuscular injection, separated by 28 days. 25% of the participants were older than 65y; 20% had comorbidities that were risk factors for severe COVID‐19. Participants represented the racial diversity of the US population. Adverse systemic events consisted of headache, fatigue, muscle and joint pain and chills – these symptoms were higher after the second than after the first vaccine injection (80% vs. 55%, placebo: 40%) and higher in younger than in older participants. Two deaths unrelated to COVID‐19 occurred in the vaccine group (one infarct, one suicide; placebo: three deaths). Allergic reactions were reported in 1.5% and 1.1% of participants in the vaccine and placebo group respectively. Until 25 November 2020, a total of 269 COVID‐19 cases were identified. From 196 symptomatic COVID‐19 cases occurring 14 days after the second injection, 11 cases occurred in the vaccine and 185 cases in the placebo group, indicating a 94% vaccine efficacy. Findings were similar for the secondary analysis, namely protection starting 14 days after the first dose (225 cases in the placebo, vs. 11 in the vaccine group). Even when counting from the first injection day until day 42, a lower infection rate was seen in the vaccine vs. control group (7 vs. 65 cases) indicating an early onset of protection. Thirty participants in the trial had severe COVID‐19; all 30 (including one death due to COVID‐19) were in the placebo group, indicating vaccine efficacy of 100% against severe disease. At the second dose visit, 39 participants in the placebo and 15 in the vaccine group had nasopharyngeal swabs positive for viral RNA by RT‐PCR without showing symptoms, suggesting a reduction in virus excretion already after the first injection. Vaccine efficacy was similar in subgroups (female/ male; with or without risk factors; between different ethnic groups), albeit somewhat lower in subjects older than 65 y, but still high (86%). While the Pfizer vaccine for the phase 3 trial had to be stored at −70°C, the Moderna vaccine was stored at 2° to 8°C in the participating clinics. Vaccine doses could be held in syringes for up to 8 hours at room temperature before administration (Baden et al., 2021).

## Reality tests in country‐wide vaccination campaigns

### Mass vaccination in Israel

A mass vaccination campaign started in Israel with the Pfizer‐BioNTech mRNA vaccine. Data from a company which insures 4.7 million patients (half of the Israeli population) were evaluated. All persons who were newly vaccinated during a 6‐week period were matched to unvaccinated controls in a 1:1 ratio. Each group comprised 596 618 persons. During follow‐up, 10 561 infections were documented by PCR, 5996 (57%) infections were symptomatic COVID‐19 illness, 369 COVID‐19 cases required hospitalization, 229 of them were severe cases of COVID‐19 and 41 cases resulted in death. When the vaccine protection evaluation counted infections from day 7 after the second injection, the vaccine effectiveness for documented infections, symptomatic illness, hospitalization and severe disease was 92%, 94%, 87% and 92% respectively. Protection was already seen at 14–20 days after the first dose, the estimated vaccine effectiveness against the abovementioned outcomes was 46%, 57%, 74% and 62% respectively. A gradual increase in vaccine effectiveness was seen over the following weeks. The estimates were consistent with similar protection rates across the different age groups: for those 65 years or older compared with younger subjects 7 days after the second dose the protection rate was 94 to 96%. Effectiveness was slightly lower among persons with higher numbers of coexisting conditions, but people suffering from obesity, diabetes or hypertension showed comparable protection rates as people without these comorbidities. For the symptomatic illness outcome, the curves of vaccinated versus unvaccinated people started to diverge at around day 12 after the first immunization. The Pfizer‐BioNTech mRNA vaccine was also clearly effective against the B.1.1.7 variant which represented 80% of the isolates during the observation period (January 2021). The vaccinees showed a reduced level of asymptomatic infections compared with controls, but this observation should be interpreted with caution since viral testing was not done on a regular basis (Dagan et al., 2021).

### Mass vaccination in England

Individuals aged ≥ 80 years living in England had a higher odds of testing positive for viral RNA in the first 9 days after vaccination with Pfizer mRNA vaccine demonstrating that they had a higher underlying risk of infection compared with younger subjects not yet in the vaccination programme. Vaccine effects were noted at 10–13 days after vaccination, reaching an effectiveness of 70% at 28–34 days after first vaccination. From 14 days after the second injection, a vaccine effectiveness of 89% was observed. With the Astra Zeneca vaccine, vaccine effects were seen starting at days 14–20 after vaccination, reaching an effectiveness of 60% after 28–34 days and further increasing to 73% protection from day 35 onwards. Data after second AstraZeneca vaccination are not yet available. On top of the protection against symptomatic disease, cases who had been vaccinated with one dose of Pfizer vaccine had a 43% lower risk of emergency hospitalization and a 51% lower risk of death. Also subjects vaccinated with one dose of AstraZeneca vaccine had a 37% lower risk of emergency hospitalization. A single dose of either vaccine is 80% effective at preventing hospitalization, and a single dose of Pfizer vaccine is 85% effective at preventing death with COVID‐19 (data for AZ not yet available due to later start of vaccination). Protection was maintained for the duration of follow‐up of 6 weeks. Both vaccines were effective against the UK variant of concern (Bernal et al., 2021).

### Mass vaccination in Scotland

The EAVE II database contains linked information on vaccination, primary care, PCR testing, hospitalization and mortality for the entire population of Scotland (5.4 million). Between December 2020 to mid‐February 2021, 35% of the population were vaccinated with the largest increase of immunization among the first priority target group (elderly subjects). AstraZeneca vaccine was the dominant vaccine in subjects older than 75 years, while the Pfizer vaccine was introduced earlier. According to UK policy, a second vaccine injection is given 12 weeks later. The authors offer the first study of COVID‐19 vaccine effects against COVID‐19 associated hospitalization for an entire nation after a single dose of vaccine. At days 28–34 after the first‐immunization, vaccine efficacy against COVID‐19 hospitalization among those receiving the Pfizer vaccine was 85% and 94% for those receiving the AstraZeneca vaccine. In subjects older than 80 years, vaccine efficacy was 81%, dissipating doubts on immune‐senescence on vaccination and on the efficacy of the AstraZeneca vaccine in older subjects, who are at the highest risk of hospitalization and death (Vasileiou et al., 2021).

### Outlook

This minireview describes an impressive progress of SARS‐CoV‐2 vaccines against symptomatic COVID‐19 disease, based on different vaccination platforms, representing classical approaches with inactivated whole viruses, but also new vaccine technologies such as mRNA vaccines that had within a year after publishing of the first SARS‐CoV‐2 genome sequence passed from design over development to production, achieving registration and mass application. Despite all feelings of satisfaction about this unexpected and unprecedent progress and high efficacy of vaccines, there is no reason for complacency. A warning sign is already flagged by a recent vaccination trial in South Africa. In this trial, 2026 young HIV‐negative adults received two doses of the AstraZeneca vaccine containing 5 × 10^10^ viral particles or placebo. Among subjects that were SARS‐CoV‐2 antibody‐negative at baseline, mild‐to‐moderate COVID‐19 disease incidence was 94 per 1000 person‐years in the placebo group compared with 73 per 1000 person‐years in the vaccine group; indicating a meagre 22% protection rate. Among at baseline SARS‐CoV‐2 antibody‐positive participants, the COVID‐19 incidence was 82 per 1000 person‐years in the placebo and 73 per 1000 person‐years in the vaccinated group indicating an even weaker vaccine efficacy of 11%. Virus from nasal swab samples of breakthrough infections was sequenced, and 95% of cases were caused by the B.1.351 (501Y.V2) variant virus. The test took place between June and November 2020. The same AstraZeneca vaccine, which showed a 75% efficacy in preventing mild‐to‐moderate COVID‐19 in South Africa before emergence of this variant virus, was now inefficient against B.1.351 variant. Severe cases were not observed in this young healthy group. The vaccine induced a good neutralizing antibody response against the previously circulating virus, but displayed a strongly reduced neutralizing antibody titre against the variant virus in two types of neutralization tests using pseudovirus and wild‐type live virus (Madhi et al., 2021). Apparently, we deal in this pandemic with an evolving virus (Brüssow, 2021), which also necessitates an evolving vaccine approach. Hopefully, the technological advances achieved in vaccinology when dealing with the SARS‐CoV‐2 virus of the current pandemic has given us the means to also cope with this problem in less time than with the yearly influenza virus vaccine adaptations.
